# Conversion of array‐based single nucleotide polymorphic markers for use in targeted genotyping by sequencing in hexaploid wheat (*Triticum aestivum*)

**DOI:** 10.1111/pbi.12834

**Published:** 2017-10-23

**Authors:** Amanda J. Burridge, Paul A. Wilkinson, Mark O. Winfield, Gary L. A. Barker, Alexandra M. Allen, Jane A. Coghill, Christy Waterfall, Keith J. Edwards

**Affiliations:** ^1^ Life Sciences University of Bristol Bristol UK

**Keywords:** wheat, genotyping by sequencing, single nucleotide polymorphism, target capture

## Abstract

Wheat breeders and academics alike use single nucleotide polymorphisms (SNPs) as molecular markers to characterize regions of interest within the hexaploid wheat genome. A number of SNP‐based genotyping platforms are available, and their utility depends upon factors such as the available technologies, number of data points required, budgets and the technical expertise required. Unfortunately, markers can rarely be exchanged between existing and newly developed platforms, meaning that previously generated data cannot be compared, or combined, with more recently generated data sets. We predict that genotyping by sequencing will become the predominant genotyping technology within the next 5–10 years. With this in mind, to ensure that data generated from current genotyping platforms continues to be of use, we have designed and utilized SNP‐based capture probes from several thousand existing and publicly available probes from Axiom® and KASP™ genotyping platforms. We have validated our capture probes in a targeted genotyping by sequencing protocol using 31 previously genotyped UK elite hexaploid wheat accessions. Data comparisons between targeted genotyping by sequencing, Axiom® array genotyping and KASP™ genotyping assays, identified a set of 3256 probes which reliably bring together targeted genotyping by sequencing data with the previously available marker data set. As such, these probes are likely to be of considerable value to the wheat community. The probe details, full probe sequences and a custom built analysis pipeline may be freely downloaded from the CerealsDB website (http://www.cerealsdb.uk.net/cerealgenomics/CerealsDB/sequence_capture.php).

## Introduction

Single nucleotide polymorphisms (SNPs) are widely used as molecular markers in genotyping and have become the marker of choice for the genotyping of hexaploid wheat (van Poecke *et al*., 2013). Several genotyping platforms are available for the screening of SNP markers, such as array‐based technologies (Wang *et al*., 2014; Winfield *et al*., 2016), and PCR‐based technologies (Allen *et al*., 2011). In addition, the use of SNP markers has meant that the employment of marker‐assisted selection (MAS) in wheat breeding programmes is now common place (Bassi *et al*., 2016).

As new technologies develop, it is essential for existing data to be interoperable between platforms. This is of particular interest in wheat breeding in which continuity is critical (Baenziger and DePauw, 2009); while a single breeding cycle may take from 10 to 12 years (Thomson, 2014), it exists as part of a continuum where new crosses are made and selected each year (Baenziger and DePauw, 2009). If data sets from different genotyping platforms can be integrated, existing data may be used and supplemented with that generated with new platforms. The ability to reuse existing data is a means to make research more cost‐effective and accessible (Leonelli *et al*., 2017).

Array‐ and KASP™‐based technologies have been extensively used (Allen *et al*., 2011; Wang *et al*., 2014; Winfield *et al*., 2016) due to their low cost per sample, high‐throughput capabilities and streamlined data analysis pipelines (Allen *et al*., 2016). However, array‐based genotyping lacks flexibility as once an array is created, the markers on that array are fixed. Arrays are also subject to an ascertainment bias related to the number of samples and criteria used in SNP detection (Albrechtsen *et al*., 2010). The fixed nature of SNPs on an array can help cross‐project comparisons as the same SNP set is used throughout. However, if additional SNPs are later required the array must be redesigned, a process that can be expensive (Thomson, 2014).

Genotyping by sequencing (GbyS) is increasingly popular due to the low cost per data point and the ability to perform simultaneous marker discovery and genotyping (Edae *et al*., 2015) without ascertainment bias (Bhat *et al*., 2016). The choice of sequencing technology and analysis pipelines can affect the selection of SNPs detected (Torkamaneh *et al*., 2016), which may complicate cross‐project comparisons.

We hypothesized that a targeted genotyping by sequencing (TGbyS) approach, employing oligonucleotide capture probes, could offer a bridge between current genotyping arrays and sequenced based genotyping technologies. Target enrichment prior to sequencing has often been used to reduce the data complexity by focusing efforts only on loci of interest (Samorodnitsky *et al*., 2015). Exome capture is well established, and perhaps the broadest means to reduce the size of the genome (Parla *et al*., 2011; Warr *et al*., 2015). While more specific techniques such as R gene enrichment sequencing (RenSeq) use target enrichment technique to focus on specific gene families, RenSeq may be used to identify SNPs within, or closely linked to R genes (Jupe *et al*., 2013), a powerful tool in the identification of disease resistance genes.

As existing target enrichment techniques successfully identify SNPs within the captured regions, we argue that by targeting areas surrounding previously characterized SNP markers we can provide a target capture probe set which will allow the resulting data to be directly comparable to previously used genotyping platforms. Used in isolation or as part of a wider target capture, the use of cross‐platform probes would allow the same set of SNPs to be genotyped across projects regardless of genotyping method facilitating the reuse and supplementation of existing data sets.

We present here the use of in‐solution, target enrichment in wheat, using capture probes currently employed in array‐based genotyping. The Axiom® and KASP™ data generated by our approach forms part of an existing publicly available data set hosted on the CerealsDB website (www.cerealsdb.uk.net; Wilkinson *et al*., 2016).

## Results

### Probe design

Mapped and polymorphic capture probes were designed based on previously validated markers as described in the Experimental procedures. Co‐dominant probes were predominantly selected as these are able to discriminate between homozygous and heterozygous states (Allen *et al*., 2013), a smaller number of dominant and partially co‐dominant were also included. While an even distribution of markers was not intended, capture probes were distributed throughout the wheat genome (Table S1). There were fewer markers located on the D genome (13.4%) compared to the A and B genomes (36.4% and 50.1%) which correlated with the reduced representation of D genome markers in the array probe set (Allen *et al*., 2016).

### Sequencing statistics

Following targeted capture and sequencing using the Illumina NextSeq platform (2 × 150 bp), 304 873 305 reads were generated from 31 wheat accessions. The number of fastq reads per variety ranged between 21 962 466 (Cadenza) and 35 853 480 (Caphorn). After trimming, between 52.49% (13 117 728 reads; Battalion) and 64.49% (17 317 486 reads; Savannah) reads remained with a uniform 35.27%–35.31% sequence quality (Table 1).

**Table 1:** The number of sequencing reads and sequencing quality score for each variety. Adaptor trimming and quality check were carried out using Sickle version 1.33 (Joshi and Fass, 2011). The reads are available from NCBI sequencing read archive (Project accession: PRJNA349252)

Variety	Total number of fastq reads	Total number of fastq reads after trimming (%)	Sequence quality before trimming	Sequence quality after trimming
Alchemy	26 027 926	15 962 990 (61.33)	34.56	35.29
Apogee	28 041 950	17 448 872 (62.22)	34.60	35.30
Avalon	26 109 364	15 868 878 (60.78)	34.58	35.29
Battalion	24 989 864	13 117 728 (52.49)	34.42	35.27
Cadenza	21 962 466	13 266 852 (60.41)	34.56	35.29
Caphorn	35 853 480	22 554 522 (62.91)	34.61	35.30
Chinese Spring	26 889 740	16 967 472 (63.1)	34.61	35.30
Claire	30 598 106	18 763 754 (61.32)	34.57	35.30
Cocoon	26 623 364	16 586 542 (62.3)	34.60	35.31
Consort	31 497 188	19 825 662 (62.94)	34.61	35.30
Cordiale	23 419 158	14 324 164 (61.16)	34.61	35.30
Evolution	23 395 860	14 035 648 (59.99)	34.57	35.30
Exsept	25 121 678	16 083 460 (64.02)	34.61	35.31
Galahad	33 196 828	19 799 898 (59.64)	34.55	35.29
Gallant	26 343 408	15 973 848 (60.64)	34.56	35.30
Gatsby	27 481 234	16 727 950 (60.87)	34.58	35.29
Glasgow	32 678 758	19 101 072 (58.45)	34.52	35.28
Hereward	24 876 424	15 346 846 (61.69)	34.59	35.30
Humber	26 320 222	16 541 152 (62.85)	34.61	35.30
Keilder	26 019 170	16 061 178 (61.73)	34.58	35.30
Mendel	29 329 778	16 059 206 (54.75)	34.46	35.28
Opata	25 953 102	15 725 162 (60.59)	34.57	35.29
Paragon	26 175 096	16 860 030 (64.41)	34.63	35.30
Recital	26 159 128	16 797 210 (64.21)	34.63	35.30
Reflection	27 332 360	17 174 174 (62.83)	34.61	35.30
Rialto	29 480 576	17 848 792 (60.54)	34.56	35.29
Robigus	26 538 010	16 468 758 (62.06)	34.59	35.30
Savannah	26 854 768	17 317 486 (64.49)	34.63	35.30
Skyfall	26 245 230	15 728 342 (59.93)	34.58	35.29
Solstice	29 430 650	18 067 354 (61.39)	34.59	35.29
Xi19	25 234 874	14 978 980 (59.36)	34.54	35.29

Over 57% (57.8%) of the sequences could be aligned to the 120 bp capture probe sequences (Table S2). A sequence alignment of 84.5% was achieved using the longer reference sequences from which the Axiom® probes were originally designed (Winfield *et al*., 2012). An aliquot of the postcapture library was also sequenced using the Illumina MiSeq (Illumina: San Diego, CA) platform (2 × 300 bp). This resulted in 78.6% of the sequences aligning to the 120 bp capture probe sequences (Table S2) and 90.8% aligning to the original reference sequence.

Using the criteria described in the Experimental procedures, we were able to generate a genotype call across all 31 accessions with 13 183 capture probes (83.9% of the original probes; Table S3). There were 187 probes which could not be used to generate a genotype for either of the capture probe pairs on any of the varieties. All 187 probe sequences were confirmed by a BLAST search of the IWGSC Whole Genome Assembly (IWGSC WGA v0.4) as present in hexaploid wheat. The 187 failed sequences contained a significantly higher (*P* < 0.0001) %GC than the total probe set, 66.13% compared with 49.34%.

**Figure 1:**
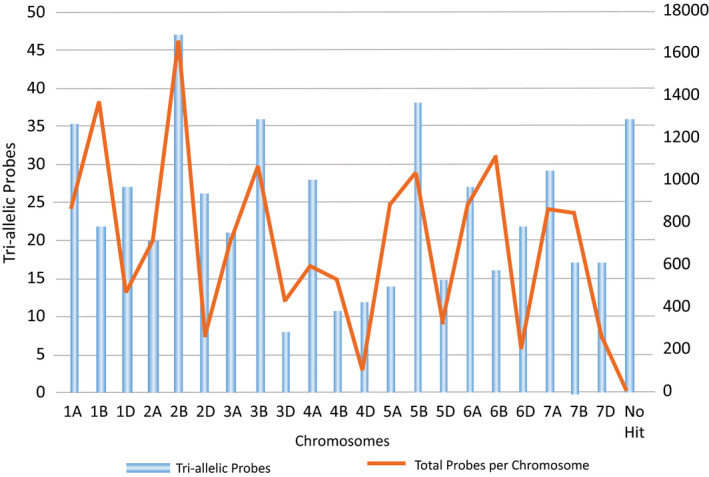
The genomic distribution of capture probes which captured a tri‐allelic single nucleotide polymorphisms in relation to the total number of capture probes used. Chromosome locations are based on IWGSC Whole Genome Assembly v0.4.

Of the 13 183 probes from which a genotype was generated, 521 appeared to be tri‐allelic; that is, there were three possible SNP genotype calls (Table S3). The distribution of the tri‐allelic probes corresponded to the total capture probe distribution (Figure 1). There were 87 SNPs which were tri‐allelic in Chinese Spring. A TBLASTX analysis was performed for these SNPs using the IWGSC WGA v0.4 Chinese Spring assembly (Table S4). The top three BLASTX results returned homoeologues of the same chromosome for 82 of the 87 SNP locations.

### Comparison to existing genotyping data

#### Axiom platform

The TGbyS data were compared to the publicly available wheat Axiom® 35K Breeders array data (Allen *et al*., 2016) hosted on CerealsDB (www.cerealsdb.uk.net) to investigate interoperability. To carry out such a comparison, only the 11 088 probes for which there was a genotype call available on both platforms were used. This included the removal of the 521 probes with a tri‐allelic genotype (Table S3) as the identification of a tri‐allelic genotype is not possible with a genotyping array. To allow comparison between sequence data (nucleotide) and Axiom® data (AA/BB score), the genotypes were converted to a numerical system as described in Experimental procedures.

**Figure 2:**
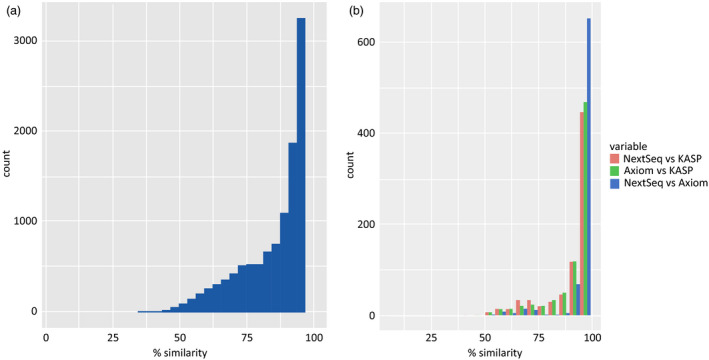
Similarity of genotype between genotyping platforms using the same probe design. The number of probes is plotted against the percentage genotype similarity when compared to the varieties (a) Comparison between TGbyS and Axiom, a bin size of 31 was used to generate the histogram. (b) Comparison between TGbyS, Axiom and KASP, a bin size of 20 was used to generate the histogram.

**Figure 3:**
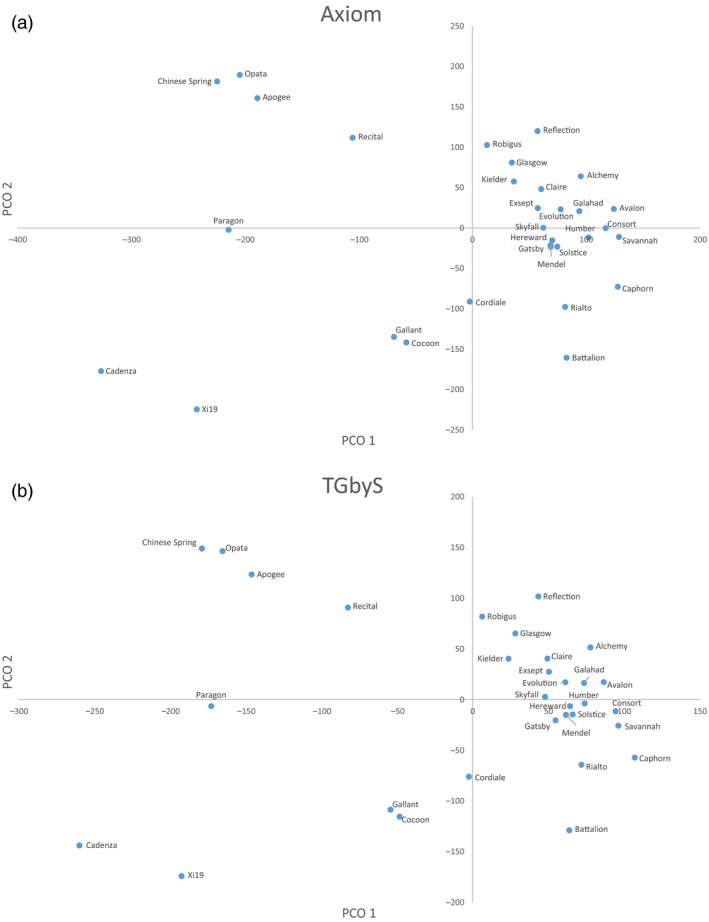
Principal co‐ordinates plot comparison of (a) Axiom genotype data and (b) TGbyS genotype data generated by NextSeq sequencing. Data generated for the 11 088 probes for which there was no missing data across 31 varieties. Clustered by squared Euclidean distance.

Similarity between the TGbyS and array‐based Axiom® data was measured across the 31 varieties for each probe (Figure 2). There were 3256 probes (29.4% of total used) with a matching genotype between platforms for at least 30 of the 31 varieties (95%). The congruence of genotypes between platforms did not appear to be linked to a particular variety. To confirm this, we examined the intervarietal patterns of genotypic variation for the 11 088 probes across all 31 varieties (Figure 3). The pattern of genotypic variation between each wheat variety was comparable between the Axiom® (Figure 3a) and TGbyS data sets (Figure 3b) when compared using a similarity matrix. There was an average 85.7% similarity across all varieties between the two platforms.

#### KASP platform

To provide an additional platform for comparison, data from the established KASP™ platform were obtained from CerealsDB (www.cerealsdb.uk.net/cerealgenomics/CerealsDB/kasp_mapped_snps.php). Of the 11 088 capture probe sequences without missing genotypes, 782 probes were also available as KASP™ data for 20 of the 31 varieties. The genotype data generated from KASP™ probes were interoperable between the TGbyS and Axiom® data sets with 566 and 588 probes, respectively, generating a matching genotype for 18 of the 20 varieties (90%; Figure 2b).

### Detection of additional ‘Off‐Target’ SNPs

Using the conservative parameters described in the Experimental procedures, SNP discovery was carried out to identify any SNPs surrounding the original target. In 287 of the captured sequences, one or more additional SNPs were detected equating to an additional 384 SNPs (Table S5). These sequences were annotated using a BLAST search (Table S6) which identified a number of annotations including several proteins associated with disease resistance. Of the original 15 167 capture probe sequences, 418 (2.8%) were annotated as disease resistance genes. This figure was greater for those probe sequences containing two SNPs (5.0%), while those containing three or more SNPs contained fivefold (15.0%) the number of sequences that were annotated as disease resistance genes. The presence of increased variability in disease resistance genes is already known (Clark *et al*., 2007), and the ability to detect these additional polymorphisms using TGbyS is advantageous.

To identify SNPs detected together on a contiguous region of sequence (In phase SNPs), different SNP detection parameters were used, as described in the Experimental procedures. This identified 7504 contigs suitable for haplotype analysis. Of these, 1697 were large contigs spanning multiple capture probes and there were 5807 contigs which only contained a single capture probe.

There was less interoperability between the Axiom® data and that of capture probe sequences with multiple additional SNPs (75.9%) compared to those without any additional SNPs (85.3%). For these SNPs, the quality of the Axiom® generated data was investigated. The Axiom® genotyping software classifies probes into quality categories depending on the performance of the probe in the tested accessions. In the original Axiom® genotyping data (Winfield *et al*., 2016), 6% of the total 15 167 probes used were classified as having a call rate below the threshold for genotyping; of the probes with more than three additional SNPs, this figure rose to 14.3%. There was only one probe with five additional SNPs and one probe with seven additional SNPs, both of which were classified as unsuitable for genotyping by the Axiom® genotyping software.

### Identification of the cross‐platform probe set

We identified 3256 probes with the greatest degree of interoperability. These ‘Cross‐Platform’ probes generated an unambiguous genotype and ≥95% similarity between the TGbyS and Axiom® genotypes. (Table S3, or from the following URL: www.cerealsdb.uk.net/cerealgenomics/CerealsDB/sequence_capture.php).

The characteristics of the cross‐platform subset differed from the total 15 167 probe set for a number of traits which may be considered for future probe design (Table 2). There was a minor but significant (*P* < 0.0001) difference in mean %GC content between the total probe set (49.39%) and the cross‐platform set (45.52%) (Figure S1). The %GC content of all 15 167 sequences was within the sequencing guidelines for both NextSeq and MiSeq sequencing protocols.

**Table 2:** Summary of probe properties between the total 15, 167 probe set and the 3256 cross‐platform probe set

	Total probe set	Cross‐platform set
%GC content	49.39	45.52
% Co‐dominant	49.74	61.5
% Dominant	28.29	18.6

**Figure 4:**
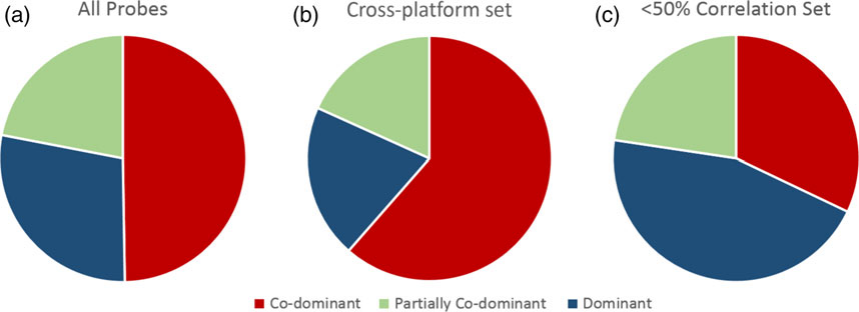
The ratio of co‐dominant, dominant and partially co‐dominant probes between subsets. (a) Total probe set. (b) Probes identified as part of the cross‐platform set. (c) Probes which generated a genotype which correlated between TGbyS and Axiom platforms for fewer than 16 of the 31 varieties (<50% correlation).

There was a higher ratio of co‐dominant probes and a reduced ratio of dominant probes in the cross‐platform subset as compared to the total 15 167 probe set (Table 2; Figure 4). The probes with the lowest interoperability (<50%) had a higher ratio of dominant probes with a reduced ratio of co‐dominant probes (Figure 4c).

**Figure 5:**
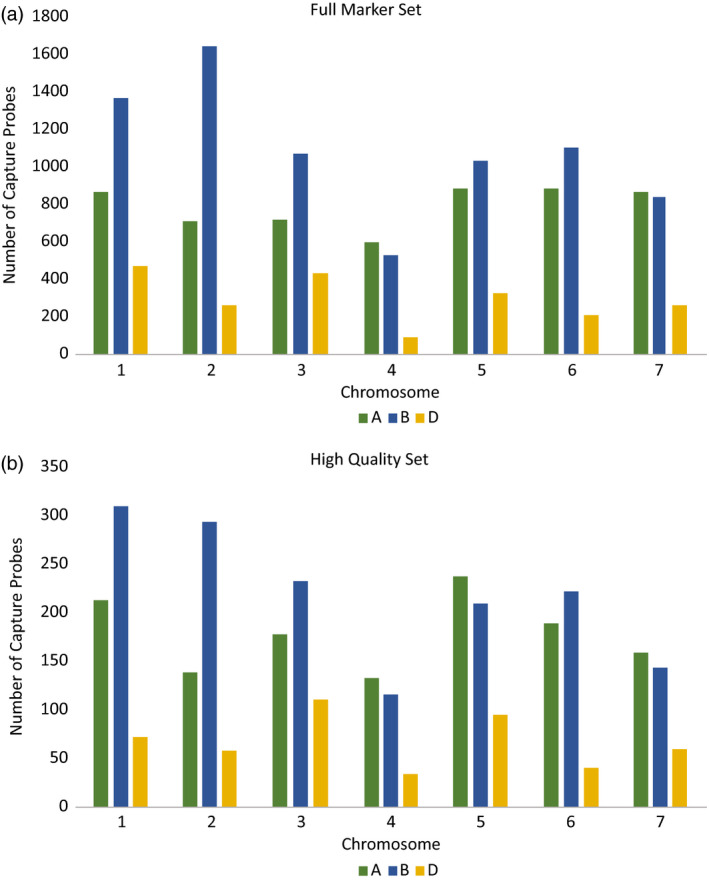
Distribution of capture probes throughout the wheat genome for full capture probe set and the cross‐platform subset. The full set represents 15 167 probes, and the cross‐platform set represents 3256 probes.

The distribution of markers throughout the genome in the cross‐platform probe subset was similar to that of the original probe set with between 15% and 38% of the original probes per chromosome (Figure 5). The chromosome with the fewest markers represented in the cross‐platform subset was 4D (34 markers), which was in proportion to the low number of markers originally mapping to this location.

The more ‘useful’ quality categories identified by the Axiom® genotyping [poly high resolution; no minor homozygote; off‐target variants (OTV)] constitute 60% of the total sequence capture probes but over three quarters of the probes in the cross‐platform subset (Table S3), probes with the highest quality Axiom® data tend to generate the best targeted genotyping by sequencing data.

## Discussion

### Sequencing statistics

The sequencing data aligned well to the reference sequence which covered regions either side of the 120 bp capture probe sequences, but did not align fully to the shorter capture probe sequences. It appeared that the target regions had been captured, but the full length of the capture probe region was not always sequenced. This was theorized to be as a result of sequence length (2 × 150 bp), as evidenced by the improved alignment of fragments sequenced with a longer read length (2 × 300 bp). It is possible that a library consisting of shorter fragments would result in a captured fragment of similar size to the capture probe. This would allow better alignment to the probe sequence for shorter read lengths; however, this is not necessary to obtain accurate genotyping data. Previous probe capture studies have shown that as long as the target SNP is captured, the sequence length covered does not affect the genotype call as much as the number of capture probes used (Holtz *et al*., 2016). The reference sequences as well as the capture probe sequences are publicly available (Winfield *et al*., 2012; Table S3).

There were only 187 probes for which a genotype could not be generated for any of the 31 varieties. These probe sequences were confirmed as present within the wheat genome indicating that the failure to capture the sequence was not due to a dissimilarity to the target sequence. As both capture probe designs of the probe pair failed in these instances, the design rather than stochastic variation is a possible cause. The problem in probe design may be related to %GC, which was higher in the failed probes. It is possible that %GC may affect the production of capture probes and is known to have an adverse effect on target capture (Bodi *et al*., 2013; Chilamakuri *et al*., 2014). The %GC of the cross‐platform probe set was also noted to be lower than the mean %GC of the full 15 167 probe set. It appears that %GC content should be a point to consider in future probe design. The majority of probes generated a genotype across all the varieties despite the genotype detection parameters described in Experimental procedures being very strict. Relaxing the stringency of the cut‐off parameters may have generated more genotypes where reads were close to the cut‐off value; however, the accuracy of the genotype calls would invariably be lower as there is a greater chance of error with less reads.

There were a small number (521) of tri‐allelic probes identified. As tri‐allelic SNPs may indicate either highly polymorphic regions of the wheat genome or copy number variants, the location of the sequences was identified in the IWGSC WGA v0.4 Chinese Spring assembly (Table S4). Of the 87 tri‐allelic sequences present in Chinese Spring, 82 seemed to exist in homoeologues of the same chromosome.

### Comparison to existing genotyping data

To be fully functional, genotyping markers on one platform should be interoperable with markers on a range of other platforms. To ensure that data can be exchanged between genotyping platforms, we examined the TGbyS data and the publicly available wheat Axiom® 35K Breeders array data (Allen *et al*., 2016) hosted on CerealsDB (www.cerealsdb.uk.net).

Capture probes were designed based on previously validated SNP markers to ensure genotyping data could be used across platforms. The genotypes generated in this study were highly correlated with the genotypes previously generated on the Axiom® and KASP platforms (Figure 2). Those with the greatest similarity were identified as part of a separate cross‐platform probe set. There were 3256 probes identified as part of the cross‐platform subset which generated an unambiguous genotype that had good interoperability with existing genotyping data. Comparison between the total probe set and the cross‐platform subset indicated characteristics that could direct the design of good quality probes in future.

The cross‐platform probes had a lower %GC compared with the total probe set, while the %GC was higher in the probes from which a genotype could not be generated. The %GC of all probes were within sequencing guidelines; however, as %GC‐rich regions are prone to mis‐pairing with other %GC‐rich regions, it is considered beneficial to reduce the number of probes with such %GC‐rich regions within a probe set. For subsequent probe designs, an average %GC of 45.5 is suggested where possible.

The cross‐platform subset also contained a higher ratio of co‐dominant probes and a reduced ratio of dominant probes compared to the original probe set (Figure 4). Detection of SNPs in hexaploid material is complicated by the presence of homoeologues. Co‐dominant probes usually only amplify a single homoeologue and so are able to distinguish between heterozygotes and homozygotes. This makes them particularly useful as markers (Allen *et al*., 2013). Dominant probes are less likely to distinguish between homozygous and heterozygous SNPs. The increased ratio of co‐dominant probes in the cross‐platform set is likely due to some of the difficulties in obtaining a genotype from a dominant probe on either platform. As co‐dominant probes are considered easier to interpret for breeding and genomic research, the presence of a higher number of co‐dominant probes in the cross‐platform probe set is advantageous.

Probes with missing data were not considered for the cross‐platform set as the ability to compare genotype was compromised. However, as data were often missing from a small number of varieties, these markers may still be particularly useful especially if present in regions of interest. Some of the capture probes, which did not correlate well with the Axiom® data, were in regions with multiple surrounding SNPs and may be particularly relevant in a breeding context.

Genotyping platforms that use cluster pattern recognition, such as arrays, rely on the intensity of two signals to ‘identify the genotype to which each sample most likely belongs’ (Affymetrix, 2011). On these platforms, where there are greater or fewer than three clusters, there is an increased error rate in genotype call (Bassil *et al*., 2015). Probes that are in variable regions with multiple SNPs can produce a nonstandard cluster pattern that is difficult to genotype algorithmically. Previously, it has been observed that an incorrect or missing genotype call can be made if uncharacterized variation exists between the sample DNA and the sequence used to design the array probes (Didion *et al*., 2012). While sophisticated genotype calling algorithms may be able to mitigate this effect in array data, we observed that an increase in the number of surrounding SNPs was associated with a reduction in suitability for the genotype calling software. In instances where a target SNP cannot be characterized by arrays, the application of TGbyS can generate more detailed and accurate information, identifying both the target SNP and the nature of the surrounding variation. This fact has been observed previously with a modified version of RenSeq (MutRenSeq) where detailed sequence variations of EMS mutants were identified only in the R genes of interest in wheat and wheat relatives as a means of identifying stem rust resistance (Steuernagel *et al*., 2016). The approach of RenSeq and TGbyS provides more detailed information than simple SNP genotyping yet have reduced sample costs and increased reproducibility compared to whole exome capture.

Additional SNPs were identified in addition to the central target SNP for 285 of the probe captured sequences. Strict parameters were used for SNP detection: a minimum of 10 aligned reads per contig; the genotype to be present in at least 20% of reads, which invariably resulted in the characterization of fewer additional SNPs than are actually present. A BLAST search revealed that the percentage of sequences annotated as coding for disease resistance proteins was greater for the sequences with additional SNPs. Disease resistance genes evolve rapidly (Keller and Feuillet, 2000), and increased variability in disease resistance genes is already known (Clark *et al*., 2007). While the probe set selected here was not intended for a disease resistance study, it indicates that the highly variable regions associated with disease are more difficult to genotype by the classical array method. For data relating to rapidly evolving regions of the genome, a sequencing approach may prove more informative.

Once a set of closely located SNPs are characterized, it should be possible to identify sets of contiguous SNPs capable of providing haplotype information. This has been found useful for more discriminative trait mapping (N'Diaye *et al*., 2017) and for transfer of data across gene mapping projects (Jordan *et al*., 2015). There were 7504 sequenced contigs found to contain at least three contiguous SNPs suitable for a haplotype study. In some instances, the haplotypes were larger than three contiguous SNPs; however, for the purpose of identifying the possibility of haplotype detection in a reduced TGbyS data set, the variations in haplotype size were not pursued.

Analysis of the TGbyS data is complex requiring significant computing requirements and bioinformatics expertise. The sequence data and alignment files generated can be extremely large, and this analysis of 31 varieties utilized more than 1.2 Tb of disk space. In comparison, a typical Axiom® genotyping project can consist of hundreds of varieties and only use around 50 Gb diskspace (approximately 4% of the diskspace required for the TGbyS analysis). Unlike the Axiom® array platform, there are currently no dedicated computational pipelines for the analysis of TGbyS data sets, so custom perl scripts were written to combine standard adaptor trimming and alignment software with genotype calling. It is anticipated that commercial or open source software will eventually become available, as GbyS becomes more popular with the research and breeding communities, which can streamline the data analysis in a comparable manner to array analysis pipelines.

There is the capacity within the protocol to reduce the sequencing cost by higher throughput library preparation and combining greater numbers of indexed samples within a single capture. Use of the more cost‐effective Nextera v3 library preparation kit (Illumina Inc., San Diego, CA) resulted in <1% of reads being mapped back to the capture probe sequences using BLAST (data not shown) compared to the 78.6% with the Illumina TruSeq library preparation. Difficulty combining Nextera library preparation with MyBaits® capture has been reported elsewhere (Nicholls *et al*., 2015), so this procedure may not be suitable for this purpose.

### Summary

We present this targeted genotyping method alongside a cross‐platform subset of markers that are considered to be of interest to breeders and researchers, due to their ability to accurately call genotypes in a number of varieties and their cross‐platform compatibility. Details are included to aid in the design of good quality cross‐platform probes from other existing probe sets.

We believe that these data will be of use to research teams looking to integrate cross‐platform genotyping data. With support from publishers and funding bodies, the amount of open access data has increased (Leonelli *et al*., 2017), although limited by the ability to integrate data between projects (Hamid *et al*., 2009). Any progress towards cross‐platform compatibility could improve the accessibility of data as genotyping technologies move forward.

While there was generally a good interoperability between the genotypes generated on the platforms used, the TGbyS method appeared to provide more accurate data in areas with higher levels of polymorphism. Rapidly evolving regions such as those associated with disease resistance may be one of the regions for which a sequencing method may be preferred, the use of TGbyS allows for interoperability between the sequence and existing genotype data.

Details of probe sequences, genotypes, haplotypes and custom perl scripts are freely available online via an interactive webtool, which allows users to select probes by subset and chromosomal location ( http://www.cerealsdb.uk.net/cerealgenomics/CerealsDB/sequence_capture.php).

## Experimental procedures

### Target enrichment capture probe design

Probe selection for conversion to MyBaits® capture probes (MYcroarray, Ann Arbor, MI) (Table S3) was based on previously detected SNP markers which were validated on both the Axiom® HD Wheat Genotyping Array (Winfield *et al*., 2016) and Axiom® 35k Breeders Array (Allen *et al*., 2016). Probes were designed for SNPs which had a mapped location and were polymorphic in UK breeding material.

A total of 30 334 biotinylated RNA capture probes were designed as 120mer exome‐specific probes around the Axiom®‐identified SNP. For each SNP, two capture probes were designed, one to capture each allele. All sequence capture probes and associated data are available online from the following URL http://www.cerealsdb.uk.net/cerealgenomics/CerealsDB/sequence_capture.php.

### Plant material

Wheat lines were grown in peat‐based soil in pots and maintained in a glasshouse at 15–25 °C with 14‐h light, 8‐h dark. Leaf tissue was harvested 6 weeks after germination, frozen in liquid nitrogen and stored at −20 °C prior to nucleic acid extraction. Genomic DNA was prepared using a phenol–chloroform extraction method (Burridge *et al*., 2017), treated with RNase‐A (QIAGEN Ltd., Manchester, UK) according to the manufacturer's instructions and purified using the QiaQuick PCR purification kit (QIAGEN Ltd).

The 31 varieties used in this study were as follows: Alchemy, Apogee, Avalon, Battalion, Cadenza, Caphorn, Chinese Spring, Claire, Cocoon, Consort, Cordiale, Evolution, Exsept, Galahad, Gallant, Gatsby, Glasgow, Gulliver, Hereward, Humber, Kielder, Mendel, Moulin, Opata, Paragon, Recital, Reflection, Rialto, Robigus, Savannah, Skyfall, Solstice, Xi 19. All varieties have been made publicly available through the Germplasm Resources Unit (www.jic.ac.uk/germplasm).

### Illumina sequencing library preparation

Genomic DNA was mechanically sheared using a UCD‐200 Biorupter (Diagenode, Holliston, MA) at 30 s on/off intervals for a total of 32 min resulting in an average fragment size of 250 bp. Fragments were purified using AMPure XP beads (Beckman Coulter Inc., Brea, CA), and size distribution confirmed using an Agilent D1000 Tape Station (Agilent Technologies Inc., Santa Clara, CA). Barcoded sequencing libraries were prepared using the TruSeq Nano gDNA HT sample preparation kit (Illumina Inc.) according to manufacturer's protocol (TruSeq Nano DNA Library Prep Protocol Guide, 2015).

### Target enrichment

In‐solution sequence capture of multiplexed sequencing libraries was carried out using the MyBaits® custom kit (MYcroarray) according to the manufacturer's protocol (MyBaits® user manual 3.01, 2015). For each variety, 100 ng of library was taken into the capture, resulting in 3100 ng total input for a 31‐plex capture. The captured DNA library was released from the capture beads and amplified for 12 cycles using Illumina P5 and P7 specific primers (Meyer and Kircher, 2010). Postcapture library size averaged 464 bp including Illumina sequencing adapters.

### High‐throughput sequencing

Capture probe enriched sequencing libraries were sequenced on both the Illumina MiSeq and NextSeq 500. A MiSeq v3 Paired End 2 × 300 bp kit and a NextSeq500 2 × 150 bp High‐Output v2 kit (Illumina) were used, respectively, with a final library concentration of 10 and 1.6 pm, respectively, which included 5% PhiX control library.

All reads are available from NCBI sequencing read archive using project accession PRJNA349252.

### Data analysis

Sequence data were quality checked and adaptor trimmed using Sickle version 1.33 (Joshi and Fass, 2011). Sequencing reads were aligned to the MyBaits® capture probe sequences using BWA aligner version 0.7.5a‐r405 (Li and Durbin, 2009), and the alignments were output as Sequence Alignment Map (SAM) files. The counts were extracted and genotypes generated using custom perl scripts. A cut‐off was set for the minimum number of reads aligned to a contig (for this analysis, it was 10) and also for percentage of reads required to call a genotype (for this analysis it was 20%), for example the read count A(20), T(80) would be assigned an AT genotype. This pipeline is available to download from the CerealsDB website (http://www.cerealsdb.uk.net/cerealgenomics/CerealsDB/sequence_capture_pipeline.php).

Axiom® data allele calling was carried out using Affymetrix Analysis Suite (version 1.1.0.616) and hexaploid wheat specific priors as described in Allen *et al*. (2016); the genotype data were downloaded from www.cerealsdb.uk.net/cerealgenomics/CerealsDB/axiom_download.php.

KASP™ data allele calling was carried out as described in Allen *et al*. (2011); the genotype data were downloaded from www.cerealsdb.uk.net/cerealgenomics/CerealsDB/kasp_mapped_snps.php.

To compare the genotype calls across sequencing, Axiom® and KASP™ data sets, it was necessary to encode the data from these different platforms to create a common information exchange reference model to achieve semantic interoperability. To facilitate data exchange between platforms, genotyping calls for each probe were ranked in order of their relative abundance into a numerical scoring system with the value ‘0’ assigned to the most prevalent genotype, a value of ‘1’ to the next most prevalent genotype. This numerical conversion was also performed on the Axiom® and KASP™ genotype calls to ensure interoperability of the data.

The comparison of genotype calls across platforms was made by the construction of a difference (distance) matrix using a custom python script. The matrix was imported into the R statistical software package version 3.3.1 (R Core Team, 2013), and principal co‐ordinates (PCO) were calculated using the classical multidimensional scaling (MDS) function, ‘*cmdscale*’. The first two PCO were plotted. The percentage similarity was derived using custom perl scripts, and these were plotted as histograms using R (version 3.2.3) and the ggplot2 package (Wickham, 2009). The percentage GC (%GC) content was calculated using a custom perl script.

BLAST version 2.2.26 was used to compare probe design sequences to the IWGSC Whole Genome Assembly (IWGSC WGA v0.4; accessed from https://wheat-urgi.versailles.inra.fr) and carry out homology searches against the embryophyta protein sequences downloaded from NCBI (www.ncbi.nlm.nih.gov). A BLAST search of IWGSC WGA v0.4 identified the sequences of all 187 failed probes as being present in the wheat genome indicating that a failure to capture the sequence was not due to a dissimilarity to the target sequence.

The identification of additional SNPs within the captured sequences was achieved using samtools version 0.1.19 for *in silico* SNP prediction. For each wheat variety sequenced, the SAM alignment files generated by BWA were converted into BAM files and indexed using samtools. The SNPs were called using the samtools mpileup program, and the resulting files were converted to variant call format (.vcf) using the bcftools view program included in the samtools package. Custom perl scripts were then used to extract novel SNPs from the .vcf files for bi‐allelic SNPs where the quality score was ≥100 and SNPs in common for each probe were identified across all 31 varieties.

Haplotype was generated by extracting intravarietal (between homoeolog) SNPs from SAM files using a custom PERL script filtering on a minimum number of two alleles, each with at least two high‐quality (PHRED >20) supporting reads. The co‐ordinates for these SNPs in the reference contigs were combined with those from the varietal SNPs to produce a set of all known variant positions in the combined data set. We then extracted the alleles at each variant position from every SAM file, keeping the allele calls phased for each individual read, using a custom PERL script. Phased haplotypes consisting of three alleles were catalogued for each variety sequenced, while haplotypes of two or less were ignored and longer haplotypes split into component overlapping windows of three SNPs.

## Supporting information


**Figure S1** Percentage GC content for all 15 167 probes (purple) and those identified as the cross‐platform subset (red).

**Table S1** Distribution of the custom designed MyBaits capture probes throughout the hexaploid wheat genome. Locations are based on the consensus map generated by Winfield *et al*. (2016).

**Table S2** Number of reads mapped to the capture probe sequence for each variety using two sequencing platforms. Read length was 2 × 150 bp for the NextSeq platform and 2 × 300 bp for the MiSeq platform.

**Table S3** Probe details and associated data. Probe names and codes refer to data available from the CerealsDB website (Wilkinson et al., 2016; www.cerealsdb.uk.net *). Genotype* data described here relates to TGbyS data.

**Table S4** Tri‐allelic probe locations. Capture probe sequences with a tri‐allelic bases detected in Chinese Spring were used to carry out TBLASTX analysis to determine location in comparison to the IWGSC WGA v0.4 Chinese Spring assembly. The top three hits are listed for each probe.

**Table S5** Capture probe sequences with one or more additional SNPs detected, the position of the additional SNPs within the capture probe and genotype data for the 31 varieties.

**Table S6** Captured sequences in which additional SNPs were detected with BLAST annotation for the sequence.
