# Supplementary figures and images for: Conversion of array‐based single nucleotide polymorphic markers for use in targeted genotyping by sequencing in hexaploid wheat (Triticum aestivum)
Source: Plant Biotechnol J. 2017 Oct 23;16(4):867–76. doi: 10.1111/pbi.12834 (PMC5866950; doi:10.1111/pbi.12834)

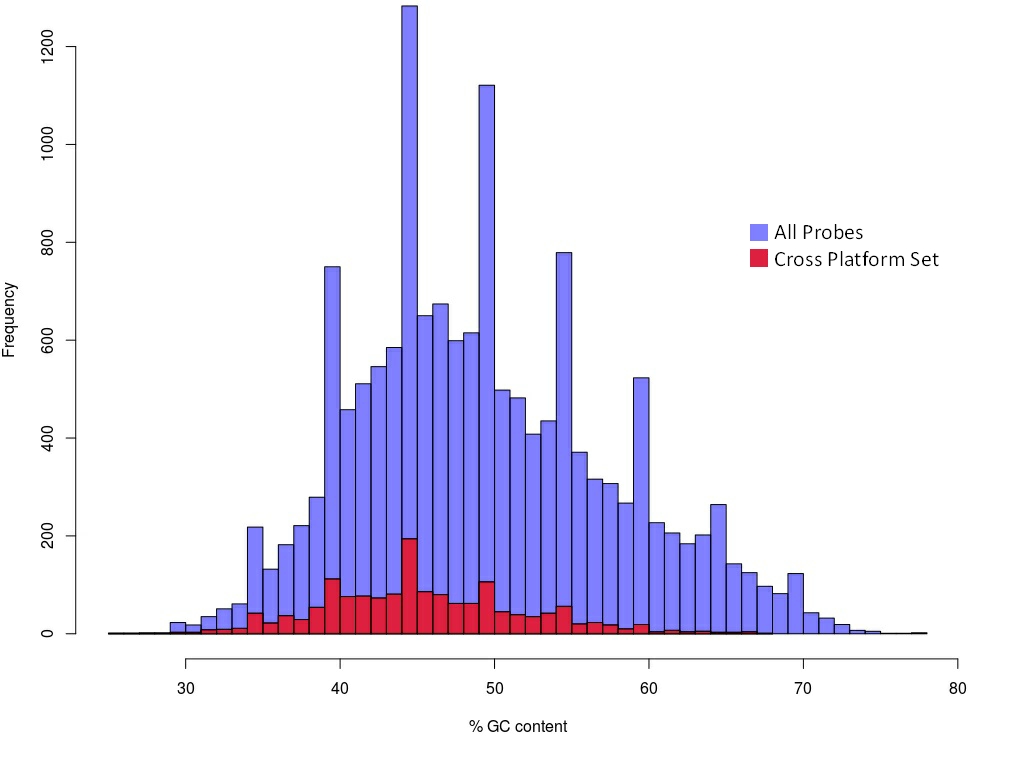

Supplement: Supplementary file 1 — Figure S1 Percentage GC content for all 15 167 probes (purple) and those identified as the cross‐platform subset (red). [file PBI-16-867-s006.jpg]
